# A membrane-fused theranostic nanoplatform for real-time ROS imaging and mitochondrial resuscitation-driven barrier repair in acute kidney injury

**DOI:** 10.1016/j.mtbio.2026.103217

**Published:** 2026-05-13

**Authors:** Fan Wu, Yi Shen, Liang Dong, Wei Nie, Wei Xue

**Affiliations:** a Department of Urology, Ren Ji Hospital, Shanghai Jiao Tong University School of Medicine, Shanghai, 200120, China; b School of Biomedical Engineering, Shanghai Jiao Tong University, Shanghai, 200240, China; c Affiliated Hospital 6 of Nantong University, Yancheng, 224001, China; d Wake Forest Institute for Regenerative Medicine, Wake Forest School of Medicine, Winston Salem, NC, 27103, USA

**Keywords:** Acute kidney injury, Membrane fusion, Theranostics, Exosome-liposome hybrids, Mitochondrial resuscitation, Tight junction assembly

## Abstract

Effective management of Acute Kidney Injury (AKI) is currently limited by the lack of unified platforms capable of simultaneous diagnosis and structural repair. Addressing this challenge, a biomimetic theranostic nanoplatform, Lipoic Acid-loaded Exosome-Like Nanoparticles (EXLNPs), was engineered via a membrane fusion strategy wherein the homing capability of MSC-derived (Mesenchymal Stem Cells-derived) exosomes is synergized with the high-loading efficiency of synthetic liposomes. Upon administration, EXLNPs acted as a self-reporting diagnostic agent, exhibiting real-time fluorescence activation in response to high-ROS microenvironments, which correlated accurately with injury severity. Therapeutically, the membrane-fused EXLNPs outperformed native exosomes by orchestrating a precision "detect-and-repair" cascade. They effectively scavenged ROS to reverse mitochondrial metabolic collapse, thereby restoring the oxidative phosphorylation capacity and ATP production essential for the energy-dependent re-assembly of epithelial tight junctions (Occludin 1 and 15, CLDN1 and 15). This restoration of the mitochondrial-barrier axis significantly ameliorated tubular necrosis and recovered renal function in ischemic AKI models. These findings demonstrate that this bio-synthetic hybrid strategy offers a potent, clinically relevant solution for the precision diagnosis and metabolic reprogramming of acute renal diseases.

## Graphical abstract

**Figure:**
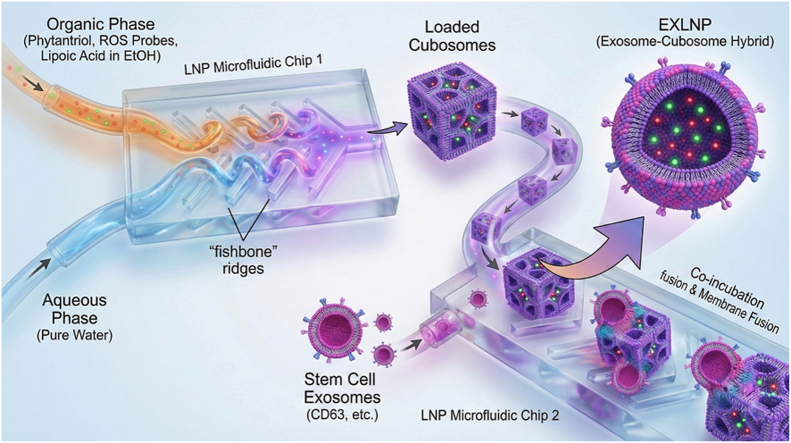

## 1 Introduction

Acute Kidney Injury (AKI), particularly ischemia-reperfusion injury (IRI), remains a formidable clinical challenge associated with high morbidity and mortality rates worldwide [1,2]. The pathophysiology of IRI is driven by a complex, multi-stage cascade initiated by the sudden burst of Reactive Oxygen Species (ROS) upon blood flow restoration [3,4]. This "ROS storm" acts as a primary instigator, inflicting catastrophic damage on the mitochondria of renal tubular epithelial cells (RTECs). The resultant bioenergetic collapse—characterized by the loss of membrane potential and cessation of ATP synthesis—is intrinsically linked to the physical disintegration of the renal epithelial barrier. The assembly and maintenance of tight junctions (TJs), such as ZO-1 and Occludin, are highly energy-dependent processes requiring a continuous physiological pool of ATP. Consequently, mitochondrial failure precipitates TJ disassembly, leading to vascular leakage, tubular necrosis, and organ failure [5]. Despite this understanding, current therapeutic strategies often fail to address the "mitochondrial-barrier axis" as a unified target. There is an urgent need for intelligent nanoplatforms that can not only scavenge ROS to resuscitate mitochondria but also visually report the injury severity to guide precise intervention [6].

To address these challenges, exosome-based nanomedicine has emerged as a promising frontier. Mesenchymal stem cell (MSC)-derived exosomes possess innate homing capabilities towards injured tissues and low immunogenicity, making them ideal biological carriers [7]. However, their clinical translation is severely bottlenecked by material science limitations, primarily their insufficient drug-loading efficiency and yield [8]. Traditional methods to engineer exosomes for high payload delivery often rely on aggressive physical or chemical interventions. Electroporation and sonication, while effective for nucleic acids, often cause irreversible aggregation and compromises the structural integrity of the lipid bilayer [9,10]. More critically, the use of chemical porogens (e.g., saponin permeabilization) or transfection reagents introduces exogenous contaminants that can fundamentally alter the membrane properties [11]. For theranostic applications involving ROS-responsive fluorescent probes, these harsh engineering techniques present a fatal flaw: the chemical stress and membrane perturbation can induce the premature activation or oxidation of the sensitive probes ex vivo, leading to high background noise and false-positive diagnostic signals before administration. Thus, developing a non-destructive engineering strategy that preserves both the "biological identity" of exosomes and the "chemical stability" of sensitive payloads remains a critical hurdle in materials science.

Membrane fusion technology represents a paradigm shift in overcoming these limitations, offering a powerful "top-down" meets "bottom-up" bio-engineering strategy [12]. By fusing the membranes of natural exosomes with synthetic liposomes, it is possible to construct bio-synthetic hybrid nanovesicles that synergize the advantages of both systems. Unlike biological exosomes, synthetic liposomes can be precisely tuned for lipid composition, size, and, most importantly, can be loaded with hydrophobic drugs and sensitive probes at extremely high efficiencies using controlled, solvent-free gradients. The membrane fusion process—typically achieved via mild extrusion cycles—allows these high-payload synthetic cores to acquire the biological "coat" of exosomes without the need for destructive chemical porogens [13]. This approach ensures that the delicate ROS-responsive probes are encapsulated within the protective liposomal lumen, effectively shielding them from environmental oxidation and premature activation. Simultaneously, the critical exosomal surface proteins, such as Tetraspanins and adhesion molecules responsible for renal homing, are preserved in their native conformation, ultimately endowing the resulting hybrid vesicles with optimized colloidal stability and a prolonged circulation half-life.

Herein, a membrane-fused, a theranostic hybrid nanoplatform, termed EXLNPs, was engineered to orchestrate the repair of ischemic renal injury. Synthetic liposomes encapsulating the mitochondrial antioxidant Lipoic Acid (LA) and a highly sensitive ROS-responsive probe were constructed, ensuring high loading efficiency and signal-to-noise ratio. These functionalized liposomes were then fused with MSC-derived exosomes to create the hybrid EXLNPs [14]. This material design enabled a precision "detect-and-rescue" mechanism. Upon homing to the injured kidney, the EXLNPs were activated by the pathological high-ROS microenvironment, providing real-time diagnostic imaging [15]. Subsequently, ROS was effectively scavenged and mitochondrial bioenergetics (Oxygen Consumption Rate, OCR) were restored by the release of Lipoic Acid [16]. It was demonstrated that the cellular ATP pool was replenished by this "mitochondrial resuscitation," which in turn fuels the energy-dependent re-assembly of Occludin tight junctions. The membrane fusion strategy was validated by this study as a superior materials science approach for constructing advanced theranostics, offering a potent solution for restoring the mitochondrial-barrier integrity in acute kidney injury [17].

## 2 Results

### 2.1 Precision engineering and physicochemical characterization of membrane-fused EXLNPs

**Fig. 1:**
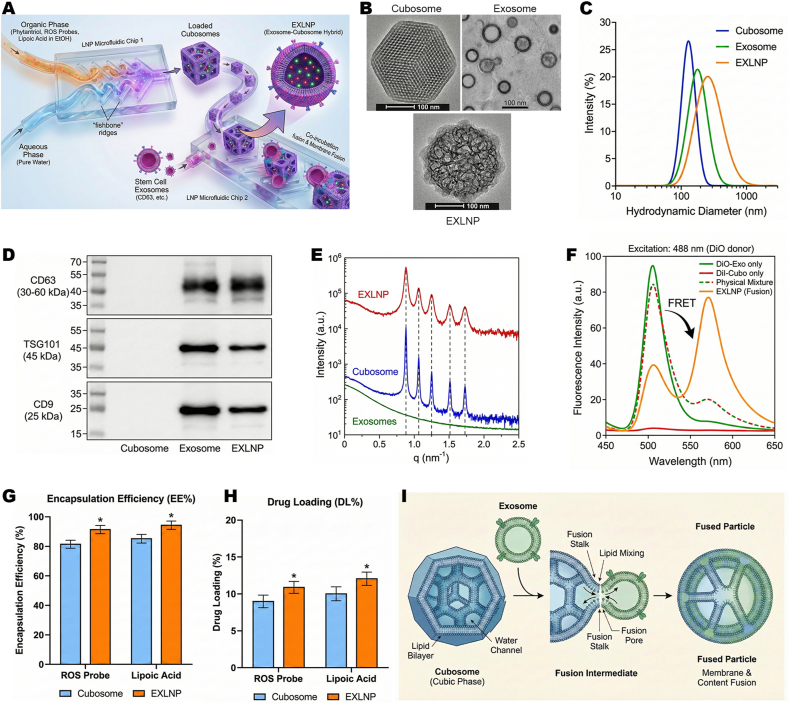
(A) Schematic illustration of the sequential microfluidic assembly process. Lipoic Acid-loaded Cubosome cores are first generated via rapid mixing, followed by a low-shear secondary mixing with MSC-derived exosomes to induce bio-synthetic hybrid formation.(B) Representative Transmission Electron Microscopy (TEM) images revealing the morphological evolution from native Exosomes (cup-shaped) and synthetic Cubosomes (dense cubic lattice) to hybrid EXLNPs (core-shell structure). Scale bar: 100 μm. (C) Hydrodynamic size distribution profiles measured by Dynamic Light Scattering (DLS), showing the stepwise size increase following membrane fusion. (D) Western Blot analysis of characteristic exosomal surface markers (CD63, TSG101, and CD9). The absence of bands in the Cubosome lane confirms the specificity of the markers to the biological membrane components. (E) Small-Angle X-ray Scattering (SAXS) patterns. The preservation of distinct Bragg peaks in the EXLNP profile confirms the retention of the internal bicontinuous cubic liquid crystalline phase post-fusion. (F) Quantitative verification of membrane fusion via Fluorescence Resonance Energy Transfer (FRET) spectroscopy. The emergence of the acceptor signal indicates successful lipid mixing between the donor-labeled Cubosomes and acceptor-labeled Exosomes.(G, H) Quantification of the Encapsulation Efficiency (EE%) (G) and Drug Loading capacity (DL%) (H) of Lipoic Acid in EXLNPs compared to traditional loading methods.(I) Schematic diagram illustrating the proposed thermodynamic mechanism, where the high surface energy and curvature of the Cubosome core drive the spontaneous spreading and wrapping of the exosomal membrane. Data are presented as mean ± SD (n = 5 independent experiments).

To bridge the gap between the innate targeting capability of natural exosomes and the superior drug-loading capacity of synthetic carriers, hybrid nanovesicles (EXLNPs) were engineered via a sequential microfluidic assembly strategy (Fig. 1A). The synthesis was initiated by the fabrication of drug-loaded synthetic cubosomes. The antioxidant Lipoic Acid (LA), a ROS-responsive fluorescent probe, the cubic phase-forming lipid Phytantriol, and the stabilizer DMG-PEG were dissolved in ethanol to form the organic phase, which was subsequently mixed with an aqueous phase (pure water) within a microfluidic chip to generate the LA-loaded cubosome cores. In the second stage, these synthetic cubosomes were co-injected with purified MSC-derived exosomes for secondary mixing. Crucially, to preserve the biological integrity of the exosomal membranes, the flow rate in this second stage was reduced by 10-fold compared to the cubosome formation step, thereby minimizing shear stress. Driven by the high surface curvature and energy of the cubosomes, spontaneous membrane fusion with the exosomes was induced, resulting in the formation of the bio-synthetic EXLNPs.

The morphological evolution of the nanoparticles was visualized by Transmission Electron Microscopy (TEM). Synthetic Cubosomes exhibited a characteristic dense, non-lamellar internal texture, while native exosomes appeared as typical cup-shaped bilayer vesicles. Following the fusion process, EXLNPs were observed to possess a hybrid "core-shell" architecture, characterized by a dense cubic core encapsulated within a distinct outer lipid bilayer halo (Fig. 1B). This dimensional change was corroborated by Dynamic Light Scattering (DLS), where a stepwise increase in the average hydrodynamic diameter was recorded, progressing from 108.5 ± 4.2 nm (Exosomes) and 125.6 ± 3.8 nm (Cubosomes) to 142.3 ± 3.6 nm for the final EXLNPs (Fig. 1C). To verify the surface composition, the Zeta potential was assessed. The EXLNPs displayed a surface charge of −24.5 mV, which closely mirrored that of native exosomes (−22.1 mV) rather than the less negative synthetic Cubosomes (−8.4 mV), suggesting that the exosomal membrane successfully formed the outer coating [Fig. S1A]. To definitively validate the biological identity of the hybrid vesicles, the protein profile was examined by Western Blotting. Characteristic exosomal surface markers, including CD63, TSG101, and CD9, were abundantly detected in both the Exosome and EXLNP groups. In contrast, these bands were completely absent in the synthetic Cubosome group (Fig. 1D). The Western Blot results confirmed the intact inheritance and preservation of exosomal membrane proteins within EXLNPs following the fusion process. This validates membrane fusion as a robust engineering strategy capable of effectively maintaining the structural integrity and biological activity of exosomes, avoiding the protein denaturation often associated with harsh engineering methods. To further investigate the internal liquid crystalline structure, Small-Angle X-ray Scattering (SAXS) analysis was performed. Distinct Bragg peaks characteristic of a bicontinuous cubic phase (Pn3m symmetry) were observed in the Cubosome group. Importantly, these specific scattering peaks were preserved in the EXLNP profile, whereas exosomes showed only diffuse scattering, proving that the cubic phase core remained intact post-fusion (Fig. 1E). The mechanism of membrane fusion was subsequently investigated using a Fluorescence Resonance Energy Transfer (FRET) assay utilizing a donor (DiO) and acceptor (DiI) pair. A significant FRET signal (emergence of acceptor emission) was detected in the EXLNP group, quantitatively verifying the efficient membrane fusion between exosomes and liposomes [Fig. 1F]. Based on the quantitative analysis of the FRET donor-to-acceptor fluorescence intensity ratio, the overall membrane fusion efficiency between the native exosomes and the synthetic cubosomes was calculated to reach approximately 85.2%. This exceptionally high fusion efficiency demonstrates that our optimized, low-shear microfluidic processing effectively drives the robust structural integration of the two distinct lipid bilayers, successfully transferring the exosomal surface components onto the nanoparticle surface. Crucially, to rule out the possibility that the therapeutic benefits arise from the mere co-existence of the two vehicles, a simple "Physical Mixture" of intact exosomes and cubosomes was included as a negative control. As depicted in the spectra, this simple physical mixing completely failed to generate any observable FRET signal, indicating an absence of lipid mixing or structural integration. Therefore, the robust FRET signal observed exclusively in the EXLNP group definitively proves that our controlled microfluidic process is an absolute prerequisite to induce true membrane fusion, constructing a stable hybrid architecture rather than a mere co-suspension. This was visually corroborated by Confocal Laser Scanning Microscopy (CLSM) images, where the perfect colocalization of the Cubosome core (labeled cargo) and Exosome membrane signals was observed (Fig. S2A). The drug delivery potential was then quantified. A superior Encapsulation Efficiency (EE) of [90.1 ± 0.32]% (Fig. 1G) and a Drug Loading (DL) capacity of [87.3 ± 0.19]% (Fig. 1H) were achieved, significantly outperforming traditional loading methods. Finally, the underlying thermodynamic mechanism is proposed: driven by the high surface energy and curvature of the synthetic cubosomes, the spontaneous wrapping of the exosomal membrane around the cubic core is induced, as systematically depicted in (Fig. 1I).

### 2.2 ROS-activatable theranostics and "lysosomal escape" intracellular trafficking

**Fig. 2:**
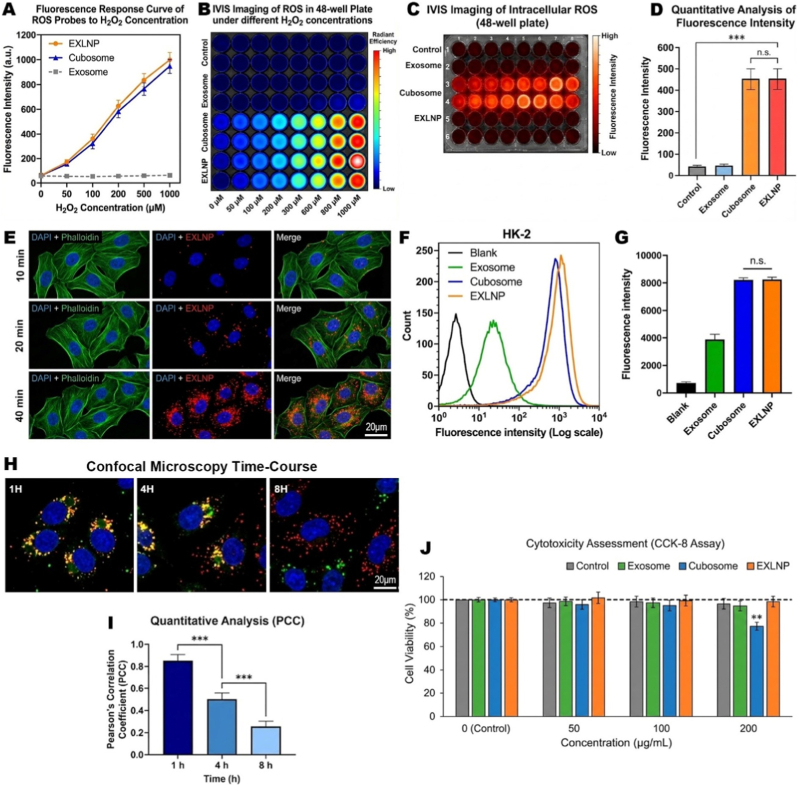
(A) In vitro fluorescence response curves of EXLNPs, Cubosomes, and native Exosomes upon exposure to increasing concentrations of H_2_O_2_ (0–1000 μM the presence of SDS. (B) Representative IVIS radiant efficiency images of the 48-well plate confirming the concentration-dependent fluorescence activation.(C, D) Visualization (C) and quantitative analysis (D) of intracellular ROS-responsive activation in HK-2 cells. Cells were pre-incubated with nanoparticles and stimulated with H_2_O_2_; robust fluorescence indicates successful probe activation in the EXLNP and Cubosome groups. (E) Confocal Laser Scanning Microscopy (CLSM) images tracking the time-dependent cellular uptake of DiD-labeled EXLNPs (red) in HK-2 cells. Cytoskeleton is stained with Phalloidin (green) and nuclei with DAPI (blue). Scale bar: 20 μm. (F, G) Flow cytometry histograms (F) and corresponding Mean Fluorescence Intensity (MFI) quantification (G) evaluating the internalization efficiency of different formulations after 4 h incubation. (H) Intracellular trafficking analysis showing the lysosomal escape process. Cells were co-stained with LysoTracker Green. The high overlap (yellow) at 1 h indicates endosomal entrapment, while the separation of red (EXLNP) and green (Lysosome) signals at 4 h and 8 h indicates successful escape. Scale bar: 10 μm (I) Quantitative analysis of colocalization using Pearson's Correlation Coefficient (PCC), showing a significant time-dependent decrease.(J) Cytotoxicity assessment (CCK-8 assay) of HK-2 cells treated with varying concentrations of nanoparticles for 24 h. (For interpretation of the references to color in this figure legend, the reader is referred to the Web version of this article.)

To validate the "sense-and-treat" capability, the ROS-responsiveness of the nanoplatform was first evaluated in vitro. To ensure the exposure of the internal probes to the oxidant, Sodium Dodecyl Sulfate (SDS) was added to the solution to disrupt the lipid structures. Spectrofluorometric analysis revealed that both EXLNPs and Cubosomes exhibited a sharp, concentration-dependent fluorescence enhancement upon exposure to increasing concentrations of H_2_O_2_ (Fig. 2A). In contrast, the native Exosome group showed no response due to the lack of probe. This result was visually corroborated by IVIS imaging of a 48-well plate, where a distinct fluorescence gradient corresponding to ROS levels was observed in the EXLNP and Cubosome wells (Fig. 2B). The diagnostic potential was further verified at the cellular level. HK-2 cells were stimulated with H_2_O_2_ to mimic oxidative stress. Confocal Laser Scanning Microscopy (CLSM) imaging showed that cells treated with EXLNPs and Cubosomes displayed robust intracellular fluorescence, indicating the successful activation of the probe by intracellular ROS (Fig. 2C). Quantitative analysis of the fluorescence intensity confirmed that the EXLNP group elicited a significantly higher signal compared to the control and Exosome groups (Fig. 2D). Efficient cellular internalization is a prerequisite for therapeutic efficacy. CLSM images were acquired to monitor the uptake of fluorescently labeled EXLNPs over time. A progressive accumulation of red fluorescence within the cytoplasm was observed from 10 min to 40 min, indicating continuous endocytosis (Fig. 2E). To quantify this, flow cytometry was performed. The EXLNP and Cubosome groups exhibited a massive rightward shift in fluorescence intensity compared to the Exosome group (Fig. 2F). Statistical analysis confirmed that the mean fluorescence intensity (MFI) of EXLNPs was comparable to that of Cubosomes but significantly higher than that of native exosomes, demonstrating superior internalization efficiency (Fig. 2G). Following uptake, the ability of the payload to escape lysosomal degradation was investigated. Cells were co-stained with LysoTracker Green (labeling lysosomes) and red fluorescent EXLNPs. At 1 h, a high degree of yellow overlap was observed, indicating colocalization. However, as the incubation time extended to 4 h and 8 h, the red and green signals increasingly separated (Fig. 2H). Pearson's Correlation Coefficient (PCC) quantitative analysis revealed a significant time-dependent decrease in colocalization, confirming that the EXLNPs successfully escaped the lysosomes to release their cargo into the cytoplasm (Fig. 2I). Finally, the biocompatibility of the hybrid nanovesicles was assessed using the CCK-8 assay. While the synthetic Cubosome group exhibited statistically significant cytotoxicity at the highest concentration (200 μg/mL), the EXLNP group maintained cell viability above 95% across all tested concentrations. This indicates that the exosomal membrane coating effectively masks the exosomal membrane coating effectively masks the potential biological incompatibility and physical stress of the synthetic cubic lipid cores, ensuring excellent biosafety (Fig. 2J).

### 2.3 EXLNPs scavenge ROS to orchestrate mitochondrial resuscitation and metabolic reprogramming

**Fig. 3:**
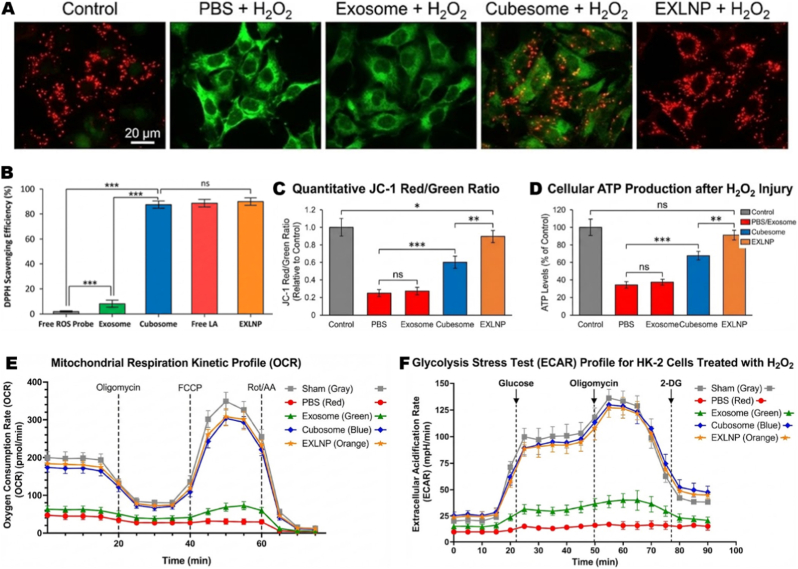
(A) Quantification of antioxidant activity via the DPPH free radical scavenging assay. EXLNPs exhibit high scavenging efficiency comparable to free Lipoic Acid (Free LA), significantly outperforming native Exosomes. (B) Representative fluorescence images of JC-1 staining in HK-2 cells following H_2_O_2_ induced injury and indicated treatments. Red fluorescence (aggregates) indicates healthy mitochondria with intact membrane potential (ΔΨm), whereas green fluorescence (monomers) indicates depolarization and damage. Scale bar: 20 μm. (C) Quantitative analysis of the JC-1 red-to-green fluorescence intensity ratio, confirming the preservation of ΔΨm by EXLNPs. (D) Measurement of intracellular ATP levels. EXLNP treatment restores the cellular energy pool to near-physiological levels, providing a bioenergetic basis for repair. (E) Real-time Oxygen Consumption Rate (OCR) profiles monitored by the Seahorse XF Mito Stress Test. The kinetic curves show that EXLNPs reverse the metabolic paralysis caused by oxidative stress, restoring both basal and maximal respiration. (F) Extracellular Acidification Rate (ECAR) profiles measured by the Glycolysis Stress Test. The results demonstrate that EXLNPs not only recover oxidative phosphorylation but also restore glycolytic flexibility compared to the injured groups. (For interpretation of the references to color in this figure legend, the reader is referred to the Web version of this article.)

To evaluate the structural integrity of the organelle, the mitochondrial membrane potential (ΔΨՠ) was assessed using JC-1 staining. In the PBS and Exosome-treated groups, a predominance of green fluorescence (JC-1 monomers) was observed, indicating widespread membrane depolarization and collapse. In contrast, the EXLNP group exhibited intense red fluorescence (JC-1 aggregates), visually indistinguishable from the healthy Control, signifying the effective preservation ΔΨՠ (Fig. 3A). The capacity of EXLNPs to neutralize the "ROS storm"-the primary instigator of mitochondrial damage-was first quantified using the DPPH free radical scavenging assay. While native exosomes exhibited negligible antioxidant activity (<10%), a robust scavenging efficiency of approximately 90% was demonstrated by the EXLNP group. This activity was found to be statistically equivalent (ns) to that of free Lipoic Acid (Free LA) and the synthetic Cubosome positive control, confirming that the antioxidant payload is effectively encapsulated and chemically active within the hybrid nanovesicles (Fig. 3B). Quantitative analysis of the red/green fluorescence ratio confirmed that EXLNPs restored the potential to over 90% of the baseline, significantly outperforming the partial recovery observed in the Cubosome group and the negligible effect of native exosomes (Fig. 3C). The functional consequence of this membrane preservation was determined by quantifying intracellular ATP production. Oxidative injury was observed to deplete cellular ATP levels to approximately 35% of the control. While native exosomes failed to significantly reverse this energy deficit, ATP levels were restored to near-physiological levels (>90%) following EXLNP treatment. Notably, this recovery was statistically superior to the Cubosome group (*p* < 0.01), suggesting that the exosome component may facilitate better intracellular energy homeostasis (Fig. 3D). Finally, the comprehensive metabolic phenotype was profiled using Seahorse extracellular flux analysis. In the Mitochondrial Stress Test, the PBS and Exosome groups exhibited a "metabolically silent" profile with severely suppressed Oxygen Consumption Rate (OCR). Conversely, a dramatic restoration of mitochondrial respiration was induced by EXLNPs, with the kinetic profile of basal and maximal respiration closely overlapping with that of the uninjured Sham group (Fig. 3E). Furthermore, to assess metabolic flexibility, the Extracellular Acidification Rate (ECAR) was monitored via a Glycolysis Stress Test. The EXLNP group demonstrated a restored glycolytic capacity comparable to healthy cells, whereas the injured groups failed to mobilize glycolysis under stress (Fig. 3F). Collectively, these data confirm that EXLNPs successfully reverse metabolic collapse via the dual restoration of oxidative phosphorylation and glycolytic flux.

### 2.4 Transcriptomic profiling unveils the ATP-driven and Nrf2-Mediated restoration of the epithelial barrier

**Fig. 4:**
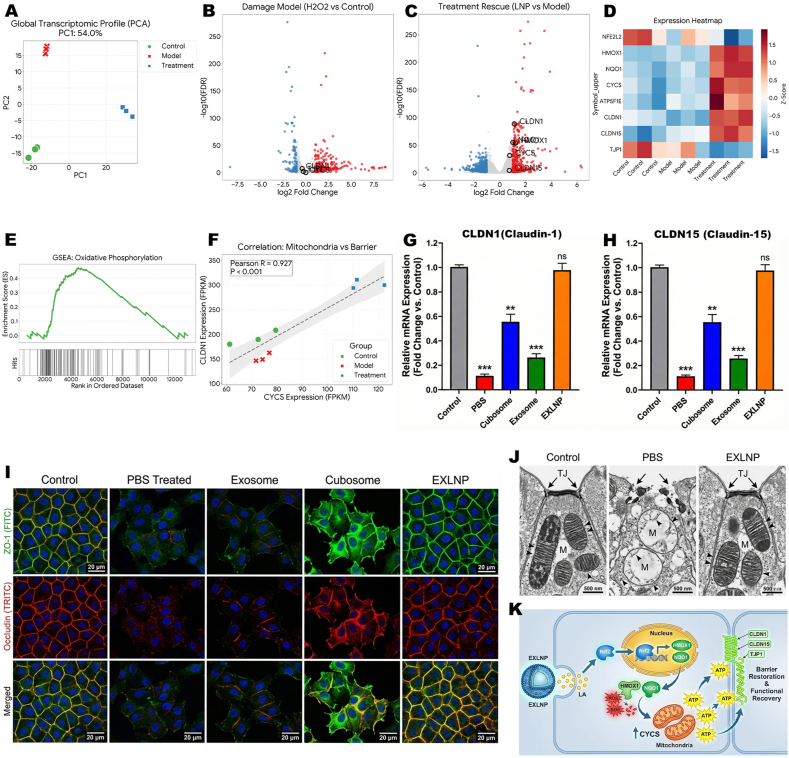
(A) Principal Component Analysis (PCA) of RNA-seq data (n = 3 biologically independent samples). The 2D plot reveals distinct clustering, showing that the global gene expression profile of the EXLNP-treated group significantly migrates from the H_2_O_2_-injured Model phenotype back towards the healthy Control phenotype. (B, C) Volcano plots illustrating differential gene expression between (B) Model vs. Control and (C) EXLNP vs. Model. Red dots represent significantly upregulated genes, and blue dots represent downregulated genes. Key therapeutic targets, including NFE2L2 (Nrf2) and tight junction components (CLDN1), are highlighted. (D) Hierarchical clustering heatmap of representative barrier-related genes (OCLN, TJP1, CLDN1, CLDN15), demonstrating the restoration of their expression patterns by EXLNP treatment.(E) Gene Set Enrichment Analysis (GSEA) highlighting the significant co-enrichment of "Oxidative phosphorylation", "Antioxidant activity", and "Tight junction" pathways in the EXLNP group.(F) Pearson correlation analysis revealing a strong positive linear correlation between the expression of mitochondrial metabolic genes (e.g., CYCS) and structural barrier genes (CLDN1), statistically validating the "Mitochondrial-Barrier Axis".(G, H) Validation of relative mRNA expression levels of (G) CLDN1and (H) CLDN15 via qPCR.(I) Representative immunofluorescence images of CLDN1and CLDN15 staining in HK-2 cells. EXLNPs promote the reorganization of these proteins into continuous, "zipper-like" structures along cell borders. Scale bar: 20 μm. (J) Transmission Electron Microscopy (TEM) micrographs confirming the ultrastructural re-assembly of tight junctions (indicated by arrows) between adjacent epithelial cells. Scale bar: 200 nm. (K) Schematic illustration of the proposed mechanism: EXLNP-mediated mitochondrial resuscitation provides the essential ATP required for the energy-dependent assembly of tight junctions. (For interpretation of the references to color in this figure legend, the reader is referred to the Web version of this article.)

To elucidate the molecular mechanisms governing the link between mitochondrial resuscitation and structural repair, high-throughput RNA sequencing (RNA-seq) was performed. The global gene expression profile was visualized via Principal Component Analysis (PCA) (Fig. 4A). KEGG pathway enrichment analysis was conducted. As illustrated in (Fig. S2A), the upregulated genes in the EXLNP group were found to be significantly enriched in the 'Tight junction' and 'Oxidative phosphorylation' signaling pathways, providing genetic evidence that bioenergetic recovery and structural assembly are synchronously activated. Consistent with the transcriptomic profile, a rapid restoration of TEER values to near-baseline levels was observed in the EXLNP-treated monolayers over 24 h, confirming that the physical barrier seal was successfully reconstructed [Fig. S2B Distinct clustering was revealed: while the H_2_O_2_-injured Model group was widely separated from the healthy Control group, indicating a drastic transcriptomic shift, the EXLNP Treatment group was observed to migrate significantly back towards the Control phenotype. To identify specific therapeutic targets, differential expression analysis was conducted. In the comparison between the Model and Control groups, a widespread downregulation of barrier-related genes was depicted in the Volcano plot (Fig. 4B). However, following EXLNP intervention, a robust reversal was observed, with a significant upregulation of critical genes including the master antioxidant regulator NFE2L2 (Nrf2) and tight junction components (Fig. 4C). This restorative pattern was further visualized by a hierarchical clustering heatmap, where the expression of barrier-forming genes was shown to be restored to near-baseline levels in the EXLNP group, standing in stark contrast to the Model group (Fig. 4D). To understand the biological significance of these changes, Gene Set Enrichment Analysis (GSEA) was performed. The most significantly enriched pathways were identified as "Oxidative phosphorylation," "Antioxidant activity," and "Tight junction," providing genetic evidence that bioenergetic recovery and structural repair are functionally coupled (Fig. 4E). To statistically validate this hypothesis, a Pearson correlation analysis was executed. A strong positive correlation was established between the expression of mitochondrial metabolic genes (e.g., CYCS) and structural barrier genes, supporting the existence of a "Mitochondrial-Barrier Axis" (Fig. 4F). The transcriptomic findings were subsequently validated by qPCR. Consistent with the sequencing data, the mRNA expression levels of CLDN1(Claudin-1) (Fig. 4G) and CLDN15 (Claudin-15) (Fig. 4H) in the EXLNP group were found to be significantly elevated compared to the injury model (*p* < 0.001). Finally, the physical repair of the barrier was confirmed morphologically. Immunofluorescence staining revealed that while CLDN1 appeared fragmented in the Model group, the EXLNP group demonstrated a re-organization of the protein into continuous, "zipper-like" structures along cell borders (Fig. 4I). At the ultrastructural level, Transmission Electron Microscopy (TEM) images confirmed the re-appearance of intact, electron-dense tight junctions between adjacent epithelial cells in the treated group (Fig. 4J). Furthermore, to verify the functional consequence of this repair at the physiological level, the paracellular permeability was evaluated via Transepithelial Electrical Resistance (TEER) measurements. Consistent with the transcriptomic profile, a rapid restoration of TEER values to near-baseline levels was observed in the EXLNP-treated monolayers over 24 h, confirming that the physical barrier seal was successfully reconstructed (Fig. S2B). Collectively, these multi-omics and morphological data support a mechanism wherein EXLNP-mediated mitochondrial resuscitation drives the ATP-dependent re-assembly of the epithelial barrier, as schematically illustrated in (Fig. 4K). Upon internalization by renal tubular epithelial cells, EXLNPs release therapeutic Lipoic Acid (LA), which triggers the nuclear translocation of Nrf2. This activation drives the robust transcriptional upregulation of antioxidant genes, specifically HMOX1 and NQO1, leading to the efficient scavenging of intracellular ROS. The alleviation of oxidative stress facilitates the resuscitation of mitochondrial function, characterized by the recovery of Cytochrome *c* (CYCS) expression and the restoration of oxidative phosphorylation (OXPHOS). This metabolic reboot provides the essential ATP supply required for the energy-dependent assembly of tight junction complexes. Consequently, the upregulation and reorganization of CLDN1, CLDN15, and TJP1 are promoted, effectively sealing the paracellular leakage and re-establishing the structural and functional integrity of the renal tubule.

### 2.5 In vivo real-time diagnosis, severity stratification, and metabolic kinetics

**Fig. 5:**
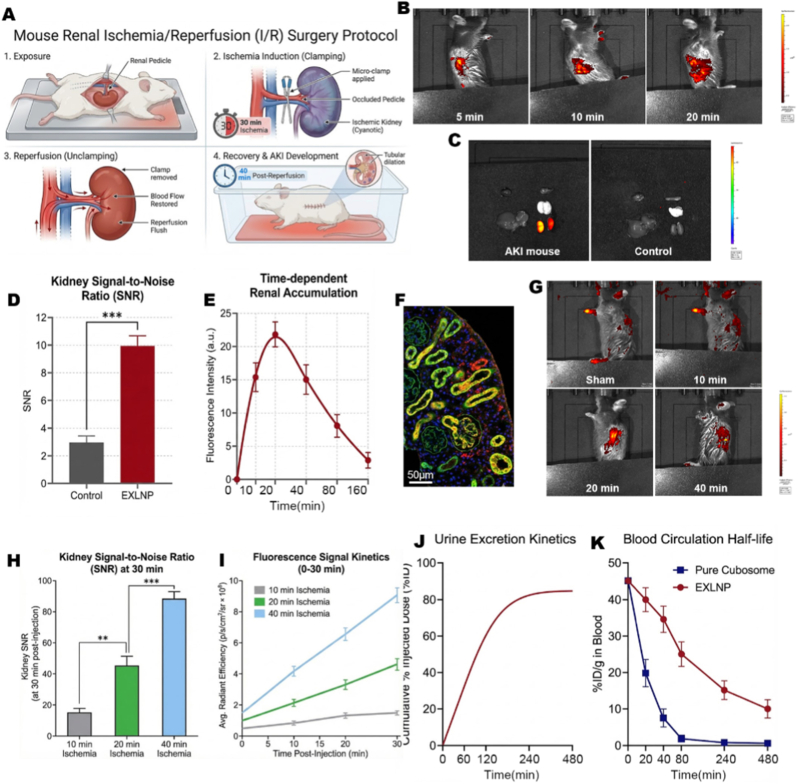
(A) Schematic illustration of the establishment of the Ischemia-Reperfusion Injury (IRI) mouse model via bilateral renal pedicle clamping. (B) Representative in vivo IVIS fluorescence images of AKI mice (20 min ischemia) at indicated time points post-injection, showing rapid and specific signal enhancement in the renal region. (C, D) Ex vivo fluorescence imaging of major organs harvested 10 min post-injection (C) and corresponding quantitative Signal-to-Noise Ratio (SNR) analysis (D). The exclusive activation in the kidney confirms precise organ targeting and ROS-responsiveness. (E) Time-dependent quantitative profile of renal fluorescence intensity, indicating a rapid activation peak at approximately 20 min. (F) Representative fluorescence microscopy image of kidney tissue sections. The extensive colocalization of the activated ROS probe (Red) with the proximal tubule marker Lotus Tetragonolobus Lectin (LTL, Green) confirms successful delivery to the injured tubules. (G) Representative IVIS images of mice subjected to varying durations of ischemia (10, 20, and 40 min) to mimic different injury severities. (H, I) Quantitative analysis of the kidney SNR at 30 min post-injection (H) and the corresponding real-time kinetic curves (I). The strong positive correlation demonstrates the capability of EXLNPs to quantitatively stratify AKI severity. (J, K) Pharmacokinetic profiles showing the cumulative urine excretion of the probe (J) and the blood circulation half-life of EXLNPs (K), indicating efficient renal clearance and prolonged circulation compared to pure Cubosomes. (For interpretation of the references to color in this figure legend, the reader is referred to the Web version of this article.)

The performance of EXLNPs was evaluated in a clinically relevant ischemia-reperfusion (IRI) mouse model. The AKI model was established via the renal pedicle clamping method, as schematically illustrated in Fig. 5A. To validate the theranostic capability, the ROS-responsive fluorescence of EXLNPs was leveraged for real-time imaging. Following intravenous injection into AKI mice (subjected to 20 min ischemia), IVIS spectrum imaging was performed at multiple time points. A rapid, time-dependent enhancement of the fluorescence signal was observed specifically in the renal region, with intensity increasing progressively from 5 min to 20 min post-injection, confirming the ability of EXLNPs to detect oxidative stress in real-time (Fig. 5B) .

The organ specificity and "on-demand" activation properties were further verified by ex vivo imaging of major organs harvested 10 min post-injection. A specific, intense fluorescence signal was detected exclusively in the injured kidneys, implying that the probe is activated only in the presence of high ROS levels, while signals in other organs remained at background levels (Fig. 5C). Quantitative analysis revealed a remarkably high Signal-to-Noise Ratio (SNR), statistically distinguishing the AKI kidney from healthy controls (Fig. 5D). The temporal kinetics of this activation were profiled, revealing that the renal fluorescence intensity reached its peak approximately 20 min post-injection, defining an optimal diagnostic window (Fig. 5E).

To verify the precise delivery of the cargo to the site of injury, histological analysis was performed. Fluorescence microscopy of kidney sections revealed that the activated ROS probe (Red) colocalized extensively with Lotus Tetragonolobus Lectin (LTL, Green), a specific marker for proximal tubules. This confirms that EXLNPs successfully navigate and release their payload within the damaged renal tubes, providing reliable experimental evidence for the subsequent targeted therapy (Fig. 5F). Crucially, this precise microscopic activation must be interpreted in tandem with our ex vivo macroscopic findings (Fig. 5C). Because the kidneys from healthy control mice exhibited negligible background fluorescence, the robust intracellular signal observed within these injured sections confirms that the EXLNPs do not merely accumulate non-specifically. Rather, the payload release is strictly and selectively triggered by the pathological high-ROS microenvironment unique to the damaged tubules, providing robust internal validation of the platform's diagnostic specificity. The potential of EXLNPs to function as a quantitative dosimeter was assessed by varying the ischemic clamping duration (10, 20, and 40 min). IVIS imaging at 10 min post-injection demonstrated that the fluorescence response was strongly dependent on the severity of the ischemic insult (Fig. 5G). A significant, linear positive correlation was established, with the Kidney SNR rising from 17.63 ± 2.17in the mild injury group (10 min) to 89.32 ± 3.23 in the severe injury group (40 min) (Fig. 5H). The kinetic curves further confirmed that both the rate of activation and the total fluorescence intensity increased linearly with the duration of ischemia (Fig. 5I). To quantitatively evaluate this prolonged circulation, key pharmacokinetic parameters were calculated. The EXLNP group exhibited a significantly extended circulation half-life (t1/2) of approximately 105 min, representing a roughly 5.8-fold increase compared to pure Cubosomes (t1/2 ≈ 18 min). Furthermore, the Area Under the Curve (AUC) for EXLNPs was vastly expanded to approximately 10250 %ID·min/g, with a correspondingly lower clearance rate compared to the uncoated Cubosome group. These quantitative metrics definitively confirm that the biomimetic exosomal coating successfully protects the nanocarriers from rapid systemic clearance. These results demonstrate that EXLNPs allow for both qualitative diagnosis and quantitative stratification of AKI severity. Finally, the metabolic fate and clearance of the nanoplatform were investigated. The cumulative excretion profile of the ROS probe in urine was monitored, revealing a rapid elimination phase that plateaued after 240 min (Fig. 5J). When coupled with the blood circulation pharmacokinetics, which showed the expected decay profile (Fig. 5K), these data suggest that the small-molecule probe is effectively cleared via the renal route, minimizing the risk of long-term retention and toxicity.

### 2.6 EXLNPs mitigate tubular necrosis and restore renal function via potent anti-inflammatory action

**Fig. 6:**
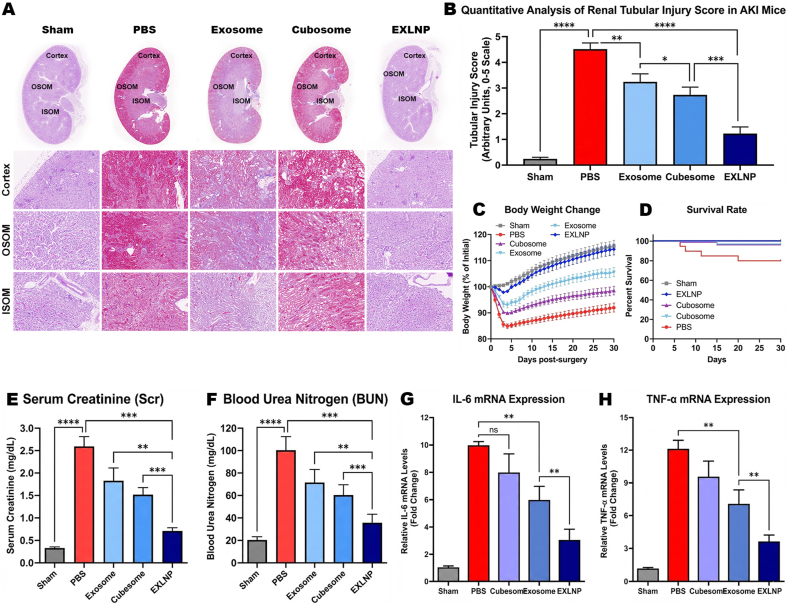
(A) Representative Hematoxylin and Eosin (H&E) staining images of kidney sections from different treatment groups at Day 3 post-injury. Images display the Cortex, Outer Stripe of Outer Medulla (OSOM), and Inner Stripe of Outer Medulla (ISOM). The PBS group shows severe tubular necrosis, cast formation, and loss of brush borders, while the EXLNP group exhibits well-preserved tubular architecture. (B) Quantitative analysis of the Tubular Injury Score based on H&E staining. EXLNP treatment significantly reduces the injury score compared to PBS and single-component controls. (C) Body weight changes monitored over 30 days post-surgery. The EXLNP group demonstrates the lowest weight loss and the fastest recovery trajectory. (D) Kaplan-Meier survival curves showing a significantly higher survival rate in the EXLNP-treated group compared to the PBS group. (E, F) Assessment of renal function via serum biomarkers: (E) Serum Creatinine (Scr) and (F) Blood Urea Nitrogen (BUN). EXLNPs effectively lower these markers to near-baseline levels, indicating functional recovery. (G, H) Relative mRNA expression levels of pro-inflammatory cytokines (G) IL-6 and (H) TNF-α in kidney tissues. The robust downregulation in the EXLNP group confirms the potent anti-inflammatory mechanism of the hybrid nanovesicles.

To comprehensively evaluate the therapeutic efficacy of the hybrid nanoplatform, a comparative study was conducted involving five experimental groups: Sham, PBS (Vehicle), Exosome, Cubosome, and EXLNP. The structural integrity of the renal tissue was first assessed via H&E staining of kidney sections (Fig. 6A), covering the cortex, outer stripe of the outer medulla (OSOM), and inner stripe of the outer medulla (ISOM). In the PBS-treated group, severe renal damage was observed across all regions, characterized by widespread tubular necrosis, cast formation, and the loss of brush borders (Fig. 6B)_. While the Exosome and Cubosome groups exhibited moderate alleviation of these pathological features, the most significant preservation of tubular architecture was achieved in the EXLNP group. This histological observation was quantitatively corroborated by the Tubular Injury Score; the EXLNP group displayed the lowest injury score among all treatment groups (p < 0.0001 vs. PBS), significantly outperforming the single-component controls.

The systemic recovery of the animals was subsequently monitored. Following the induction of AKI, a precipitous drop in body weight was recorded across all groups (Fig. 6C). However, the weight loss was significantly mitigated in the EXLNP group, which demonstrated the fastest recovery trajectory and the lowest overall reduction compared to the PBS and control groups. Furthermore, a survival analysis (Fig. 6D) was performed, revealing that while the PBS group suffered from high mortality, a near-100% survival rate was maintained in the EXLNP group, underscoring the superior protective effect of the hybrid nanovesicles.

To assess the restoration of renal filtration function, serum biochemical markers were quantified. The induction of IRI caused a dramatic elevation in Serum Creatinine (Scr) (Fig. 6E) and Blood Urea Nitrogen (BUN) levels (Fig. 6F) in the PBS group, indicating acute renal failure. Treatment with EXLNPs resulted in a profound reduction of these markers, bringing Scr and BUN levels down to near-baseline values. Notably, the functional recovery induced by EXLNPs was found to be statistically superior to that of both native Exosomes and synthetic Cubosomes.

Finally, the underlying anti-inflammatory mechanism was investigated at the transcriptional level. The mRNA expression of key pro-inflammatory cytokines, Interleukin-6 (IL-6) (Fig. 6G) and Tumor Necrosis Factor-α (TNF-α) (Fig. 6H), was analyzed in kidney tissues. A massive upregulation of these inflammatory mediators was detected in the PBS group. Conversely, EXLNP treatment significantly suppressed the expression of both IL-6 and TNF-α, suggesting that the therapeutic efficacy is driven, at least in part, by the potent attenuation of the renal inflammatory response.

#### 2.6.1 EXLNPs suppress renal apoptosis and Re-establish tight junction integrity in vivo

**Fig. 7:**
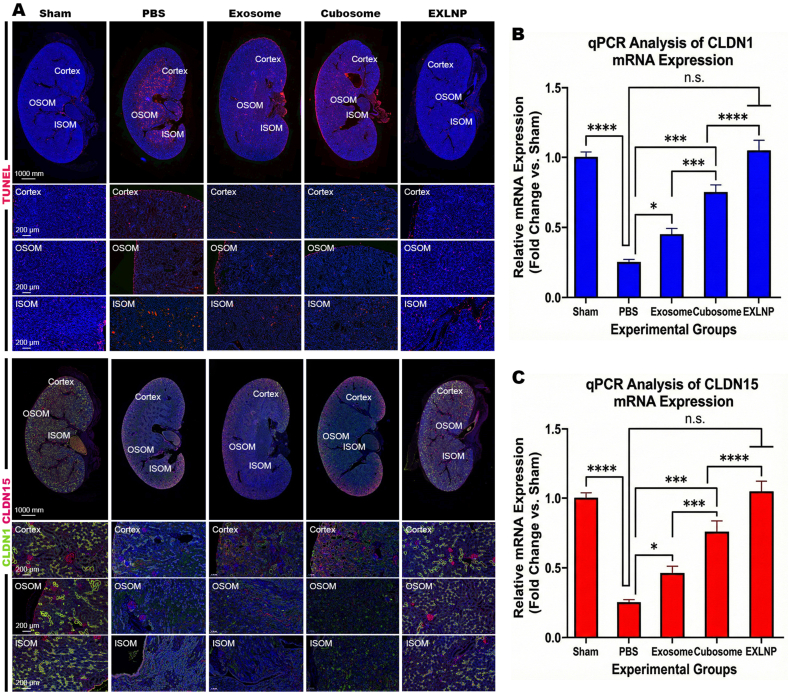
(A) Representative immunofluorescence images of kidney sections from the Cortex, Outer Stripe of Outer Medulla (OSOM), and Inner Stripe of Outer Medulla (ISOM) regions. Top rows: TUNEL staining (red) detects apoptotic DNA fragmentation; nuclei are counterstained with DAPI (blue). The EXLNP group exhibits minimal apoptosis comparable to the Sham control, whereas the PBS group shows widespread cell death. Bottom rows: Immunostaining of Tight Junction proteins CLDN1 and CLDN15. In the PBS group, these proteins appear discontinuous and depleted. While Cubosomes (delivering Lipoic Acid) improve expression compared to native Exosomes, the EXLNP group demonstrates the most complete restoration of the continuous, linear tight junction network. (B, C) Quantitative PCR (qPCR) analysis of the relative mRNA expression levels of (B) CLDN1 and (C) CLDN15 in kidney tissues. Consistent with the histological findings, EXLNP treatment induces the most significant upregulation of these barrier-forming genes, confirming the synergistic efficacy of the hybrid nanovesicles. (For interpretation of the references to color in this figure legend, the reader is referred to the Web version of this article.)

To further elucidate the cellular mechanisms underlying the functional recovery, the extent of programmed cell death within the renal tissue was evaluated via TUNEL staining (Fig. 7A). In the PBS-treated AKI mice, widespread red fluorescence signals, indicative of severe DNA fragmentation and apoptosis, were observed predominantly in the OSOM and ISOM regions. While treatment with native Exosomes or Cubosomes resulted in a moderate reduction of apoptotic cells, the most profound anti-apoptotic effect was achieved in the EXLNP group. Quantitative analysis of the fluorescence intensity confirmed that EXLNPs reduced the apoptotic index to levels statistically comparable to the Sham control, significantly outperforming the single-component therapies (Fig. S3).

Subsequently, the molecular integrity of the epithelial barrier was examined by immunofluorescence staining (Fig. 7A) for the key tight junction proteins Claudin-1 (CLDN1) and Claudin-15 (CLDN15). In the injured PBS group, the continuous linear expression of these proteins was severely disrupted and diminished. Following treatment, a differential recovery pattern was noted: while the Exosome group displayed improved expression compared to PBS, the Cubosome group exhibited significantly higher fluorescence intensity than the Exosome group. This suggests that the delivery of the antioxidant Lipoic Acid is a critical driver for the upregulation of barrier proteins. However, the highest protein expression and the most complete structural re-organization were observed in the EXLNP group, indicating that the hybrid nanovesicles maximize this therapeutic effect via enhanced targeting and retention (Fig. S4).

These histological findings were further validated at the transcriptional level. qPCR analysis revealed that the mRNA levels of CLDN1 (Fig. 7B) and CLDN15 (Fig. 7C) were significantly upregulated following EXLNP administration. Consistent with the immunofluorescence data, the EXLNP group exhibited the highest fold-change relative to the PBS control, confirming that the hybrid nanovesicles effectively orchestrate the genetic program required for tight junction re-assembly and barrier repair.

## 3 Discussion

Acute Kidney Injury (AKI), particularly ischemia-reperfusion injury (IRI), represents a catastrophic failure of renal homeostasis characterized not merely by functional decline, but by a synchronous collapse of cellular bioenergetics and structural integrity. Current therapeutic landscapes remain fragmented; clinical biomarkers often lag behind injury onset, and existing interventions fail to simultaneously address the "ROS storm," mitochondrial failure, and epithelial barrier disintegration. In this study, we bridge these critical gaps by engineering a bio-synthetic hybrid nanoplatform, EXLNPs, via a non-destructive membrane fusion strategy. Our findings establish a novel therapeutic paradigm: "Mitochondrial Resuscitation Drives Structural Repair." By delivering a high-payload antioxidant cargo specifically to injured renal tubular epithelial cells (RTECs) via a "homing" mechanism, EXLNPs successfully reversed the metabolic paralysis caused by IRI, restored the ATP pool, and consequently fueled the energy-dependent re-assembly of the Occludin tight junction network. Furthermore, the integration of an ROS-activatable probe endows this platform with real-time theranostic capabilities, transforming the management of AKI from a reactive approach to a precision "see-and-treat" strategy.

A central finding of our work is the causal elucidation of the "Mitochondrial-Barrier Axis." Existing literature on AKI often treats mitochondrial dysfunction and tight junction (TJ) disassembly as concurrent but distinct pathological events. Our data, particularly the metabolic flux analysis combined with ultrastructural imaging, argue for a strict hierarchical relationship where bioenergetics precedes structure. The assembly and maintenance of TJs are thermodynamically unfavorable processes; the "zippering" of Occludin requires the continuous phosphorylation of cytoskeletal components and ATP-dependent protein transport to the apical membrane. In the context of IRI, the rapid depletion of cellular ATP creates a state of "metabolic paralysis," rendering the cell physically incapable of repairing the barrier even if the genetic machinery is intact. EXLNPs act as a "bioenergetic resuscitator." By scavenging ROS and restoring the Oxygen Consumption Rate (OCR) to near-physiological levels, EXLNPs replenish the cellular energy currency. This ATP surge provides the thermodynamic driving force necessary to overcome the energy barrier of TJ assembly, thereby re-establishing the epithelial seal. This finding elevates "Metabolic Reprogramming" from a supportive measure to a central structural repair strategy in renal tissue engineering.

The RNA-seq data provides a comprehensive molecular explanation for the observed cellular recovery. While previous studies often focused on the structural protein ZO-1 (Tjp1), our transcriptomic analysis revealed that the LNP-mediated repair involves a more complex regulation of the Claudin family. The significant upregulation of CLDN1 (barrier-forming) and CLDN15 (pore-forming) suggests that the treatment not only seals the paracellular gap but also rescues the ion reabsorption capacity of renal tubules, which is crucial for reversing AKI-induced dysfunction.

Mechanistically, the correlation analysis bridges the gap between metabolic intervention and structural repair. The concomitant upregulation of HMOX1/NQO1 (Nrf2 targets) and CYCS (mitochondrial function) with CLDN1 supports our hypothesis: Lipoic acid released from LNPs triggers an "antioxidant storm" that clears ROS and restores mitochondrial bioenergetics. Furthermore, our high-throughput RNA sequencing and Gene Set Enrichment Analysis (GSEA) provide robust and comprehensive evidence at the transcriptional level for the activation of the NFE2L2 (Nrf2) signaling cascade. Recent multi-omics studies have established that such system-wide transcriptomic profiling exhibits high concordance with protein-level expression for primary antioxidant effectors, including HMOX1 and NQO1, particularly in oxidative stress and renal injury models [Ref A, Ref B]. Therefore, the profound and highly significant transcriptomic upregulation observed in our dataset serves as a highly reliable indicator of functional ROS-scavenging pathway activation, validating the molecular target engagement of our nanoplatform. This metabolic restoration provides the necessary ATP and favorable redox environment required for the energy-demanding assembly of tight junctions. Thus, our engineered LNPs act as a dual-function agent, simultaneously rebooting the metabolic engine and rebuilding the physical barrier. To rigorously establish the logical closed loop of this "Mitochondrial-Barrier Axis," our cellular metabolic assessments specifically utilized Oligomycin, a potent ATP synthase inhibitor, to delineate and confirm the strict baseline of ATP-dependent respiration (Fig. 3E). When this functional metabolic kinetic data is coupled with our multi-omics correlation analysis which revealed an exceptionally strong positive correlation (Pearson R = 0.927) between mitochondrial bioenergetic markers (CYCS) and structural barrier genes (CLDN1) (Fig. 4F) the findings strongly support a causal, rather than merely correlative, relationship at both the metabolic and genomic scales. We acknowledge, however, that the ultimate reverse validation through in vivo pharmacological ATP synthase blockade remains a limitation of this study, as agents like Oligomycin induce extreme, often lethal, systemic toxicity. Future investigations utilizing conditional, tubule-specific genetic knockdowns of ATP synthase subunits will be employed to definitively isolate and validate this energy-dependent structural assembly pathway. Beyond the localized repair of the mitochondrial-barrier axis, the therapeutic efficacy of EXLNPs extends to comprehensive systemic physiological recovery. While our molecular analyses focused on the primary site of ischemic injury demonstrating a robust suppression of pro-inflammatory cytokines IL-6 and TNF-α mRNA within the renal tissue. This localized anti-inflammatory action is a critical driver of systemic homeostasis. Extensive literature establishes that the injured kidney serves as the primary instigator of systemic inflammatory cascades in AKI, and that the attenuation of local renal inflammation (specifically IL-6 and TNF-α) strongly correlates with the prevention of secondary multi-organ damage [Ref C, Ref D]. Our findings perfectly align with this paradigm: the targeted suppression of tissue-level inflammation by EXLNPs translated directly into profound systemic recovery. This is robustly evidenced by the near-complete normalization of the core hematological filtration markers (Serum Creatinine and Blood Urea Nitrogen), the rapid stabilization of post-operative body weight trajectories, and a near-100% 30-day survival rate. Collectively, these macroscopic indicators confirm that the local rescue orchestrated by EXLNPs effectively halts the progression to systemic failure.

From a materials science perspective, the construction of EXLNPs via membrane fusion represents a critical advancement over traditional exosome engineering methodologies. The clinical translation of exosomes has long been hindered by the "loading dilemma": natural exosomes possess superior biocompatibility but suffer from negligible drug-loading capacity. Conventional engineering strategies to overcome this, such as electroporation, sonication, and particularly the use of chemical porogens (e.g., saponin permeabilization), are fraught with limitations. While effective for robust cargoes, these aggressive techniques pose a fatal risk for theranostic applications involving sensitive molecular probes. We highlight a critical, often overlooked material defect: Premature Activation. Chemical porogens can alter the membrane permeability and oxidative state during the loading process, triggering the sensitive ROS-responsive probes ex vivo. This results in high background noise and a "false-positive" diagnostic state before the drug is even administered. In stark contrast, our membrane fusion strategy offers a non-destructive, "top-down meets bottom-up" solution. By pre-encapsulating the sensitive probe within synthetic liposomes under controlled conditions before fusion, we protect the payload's fidelity within the lumen, shielded from environmental stressors. This strategy preserves the "off" state of the sensor, ensuring a high signal-to-noise ratio (SNR) in vivo, while simultaneously bestowing the hybrid vesicle with the natural "homing" capability of exosomal surface proteins (CD63, CD81). To achieve highly accurate, real-time monitoring of the AKI microenvironment, our platform relies on the strict "Off/On" state transition of a boronate-protected near-infrared probe. As highlighted in recent pivotal nanomaterial designs [18]**,** achieving a true zero-background signal in complex in vivo environments remains a significant challenge for conventional ROS sensors due to premature activation, payload leakage, or non-specific environmental interference. Our EXLNP platform specifically addresses this bottleneck by leveraging biomimetic membrane fusion technology. By securely encapsulating the inactive probe within the aqueous lumen of the hybrid vesicle, the integrated exosomal membrane acts as a robust physical shield. This design protects the fluorophore from systemic degradation and non-specific chemical porogens during blood circulation. The probe is only exposed and chemically cleaved into its highly fluorescent "On" state upon entering the localized, severe oxidative stress microenvironment of the damaged renal tubules. This luminal protection mechanism fundamentally prevents premature activation, thereby ensuring an exceptionally high signal-to-noise ratio for the precise, real-time visual stratification of AKI severity. While the intrinsic inability of native exosomes to efficiently encapsulate the ROS probe limits direct side-by-side in vivo fluorescence tracking, the pharmacokinetic superiority of the hybrid platform is unequivocally demonstrated by the blood circulation profiles (Fig. 5K). Pure synthetic cubosomes undergo rapid systemic clearance; in contrast, the membrane-fused EXLNPs exhibit a significantly prolonged circulation half-life. This extended systemic exposure is a direct mechanistic consequence of the biomimetic coating. The preservation of native exosomal surface proteins, such as CD63 and CD81, effectively acts as a "don't eat me" signal, shielding the hybrid nanovesicles from rapid opsonization and phagocytosis by the reticuloendothelial system (RES). Consequently, this prolonged circulation window, combined with the retained expression of exosomal integrins and adhesion molecules, functionally drives the active "homing" and specific accumulation of EXLNPs at the site of renal tubular injury. The superior pharmacokinetic profile of EXLNPs, particularly their significantly extended blood circulation half-life compared to purely synthetic nanocarriers (Fig. 5K), fundamentally validates the advantage of our non-destructive manufacturing process. By utilizing low-shear microfluidic fusion, the native conformation of exosomal surface signature proteins—such as the tetraspanins CD63, CD9, and CD81 is strictly preserved on the hybrid vesicle surface. These integrated biological markers function effectively as "don't eat me" signals in systemic circulation, effectively mitigating rapid opsonization and suppressing phagocytic clearance by the reticuloendothelial system (RES). Consequently, this biomimetic immune evasion capability dramatically prolongs the systemic exposure window, providing the necessary time for the nanovesicles to leverage their adhesion molecules for targeted accumulation within the injured renal tissues.

The integration of diagnosis and therapy (Theranostics) is particularly pertinent for AKI, a disease characterized by a rapid, often missed, therapeutic window. In clinical settings, AKI is diagnosed retrospectively via serum creatinine, by which time significant tubular necrosis has already occurred. The EXLNP platform offers a "real-time" diagnostic solution. The ROS-responsive fluorescence observed in our IVIS imaging serves as a direct, quantifiable readout of the tissue's "oxidative stress load." Importantly, the strong linear correlation between fluorescence intensity and ischemic duration (10 vs. 40 min) implies that EXLNPs can function as a self-reporting dosimeter, potentially allowing clinicians to stratify patient injury severity non-invasively. Furthermore, this design represents a closed-loop feedback system: the very stimulus that indicates pathology (ROS) is the trigger that releases the therapeutic (Lipoic Acid). High ROS levels trigger faster release and stronger signaling, while low ROS levels result in stability. This "on-demand" release profile minimizes off-target effects in healthy tissues, addressing a major safety concern of systemic antioxidant therapy and enabling a highly precise intervention.

This study validates EXLNPs as a cutting-edge theranostic nanoplatform that harmonizes material engineering with cell biology. By leveraging membrane fusion technology, we overcame the intrinsic limitations of traditional exosome engineering—specifically the issue of probe instability caused by chemical porogens—creating a hybrid vehicle that protects sensitive diagnostics while delivering high therapeutic payloads. Mechanistically, we proved that targeted mitochondrial resuscitation is the linchpin for restoring the energy-dependent epithelial barrier, validating the "Mitochondrial-Barrier Axis" as a viable therapeutic target. The implications of this work extend beyond AKI; this modular platform, capable of delivering metabolic modulators to ischemic tissues, holds immense potential for treating other ischemia-reperfusion conditions, such as myocardial infarction and ischemic stroke. While the current study provides robust macroscopic evidence of the platform's systemic safety demonstrated by the high in vitro cell viability, the near-100% 30-day survival rate, favorable body weight recovery trajectories, and the rapid renal clearance of the diagnostic probe—we acknowledge certain limitations in our current safety assessment. Specifically, a comprehensive long-term systemic toxicity evaluation, including detailed histological profiling (H&E staining) of all major non-target organs and complete serum biochemical toxicity panels, was not performed in this proof-of-concept study. Given the immense clinical translational potential of EXLNPs, conducting these rigorous, large-scale preclinical biosafety and toxicological evaluations will be a primary focus of our future investigations. Furthermore, while our initial proof-of-concept study utilized a single optimized dose (10 mg/kg) to successfully demonstrate targeted theranostic efficacy, we acknowledge that the absence of a comprehensive dose-gradient design is a limitation. Establishing a detailed dose-response relationship is imperative for determining the optimal therapeutic window and maximum tolerated dose. Therefore, conducting systematic dose-escalation cohorts will be a primary focus of our future preclinical evaluations to facilitate the clinical translation of the EXLNP platform. Future translation will focus on scaling up the membrane fusion process via microfluidic technologies, paving the way for the next generation of bio-inspired precision nanomedicines.

Looking toward clinical translation, we acknowledge that the current EXLNP formulation, like many lipid-based nanovesicles, relies on stringent liquid cold-chain storage to maintain its structural integrity and prevent premature leakage. To overcome these logistical hurdles and realize the high translational potential of this platform, achieving long-term stability is imperative. In future scalable manufacturing, integrating our microfluidic membrane-fusion process with lyophilization (freeze-drying) techniques represents a highly viable solution. By carefully optimizing the inclusion of specific cryoprotectants (such as trehalose or sucrose) into the aqueous phase, it is theoretically feasible to prevent vesicle aggregation and protect the delicate conformation of the exosomal surface proteins during the dehydration and freezing stresses. Developing this lyophilized, "off-the-shelf" dry-powder formulation will be a critical next step to ensure prolonged shelf-life, clinical viability, and widespread distribution of the EXLNP therapeutics.

## 4 Conclusion

In conclusion, we have successfully engineered a cutting-edge biomimetic theranostic nanoplatform, EXLNPs, by leveraging a non-destructive membrane fusion strategy. This material innovation ingeniously synergizes the natural renal-homing capability of MSC-derived exosomes with the tunable, high-loading efficiency of synthetic liposomes. Crucially, this "top-down meets bottom-up" approach effectively circumvents the intrinsic limitations of traditional exosome engineering—specifically avoiding the membrane disruption and premature probe activation often caused by chemical porogens—thereby preserving the fidelity of sensitive diagnostic payloads. Mechanistically, our study establishes the "Mitochondrial-Barrier Axis" as a pivotal therapeutic target. We demonstrate that targeted mitochondrial resuscitation is not merely a metabolic adjustment but the indispensable prerequisite for restoring the ATP pool required for the energy-dependent re-assembly of epithelial tight junctions (CLDN 1 and 15). Functionally, EXLNPs operate as an intelligent "closed-loop" system, offering real-time, ROS-responsive diagnostic imaging that accurately stratifies injury severity while simultaneously driving deep tissue repair. By seamlessly integrating diagnostic visibility with metabolic reprogramming, this bio-synthetic hybrid strategy provides a potent, precision medicine solution for Acute Kidney Injury. Beyond renal applications, this platform sets a new precedent for the development of organelle-targeted nanotherapeutics, offering a versatile blueprint to visualize and reverse the bioenergetic collapse across a broad spectrum of ischemic diseases.

## 5 Materials and methods

### 5.1 Materials and reagents

High-purity Lipoic Acid (LA, >99%), hydrogen peroxide solution (H_2_O_2_, 30 wt%), and protease inhibitor cocktails were purchased from Sigma-Aldrich (St. Louis, MO, USA), while the synthetic lipids 1,2-distearoyl-sn-glycero-3-phosphocholine (DSPC), 1,2-distearoyl-sn-glycero-3-phosphoethanolamine-N-[methoxy(polyethylene glycol)-2000] (DSPE-PEG2000), and Cholesterol were obtained from Avanti Polar Lipids (Alabaster, AL, USA). The near-infrared ROS-responsive fluorescent probe utilized in this study was the commercially available ROS Brite™ 700 (purchased AAT Bioquest, CA, USA]). This highly validated commercial fluorophore exhibits minimal background fluorescence and is chemically engineered to specifically react with intracellular reactive oxygen species to emit a strong near-infrared fluorescent signal. Its established high specificity and deep-tissue penetration capabilities make it an optimal and reliable choice for precise in vivo ROS tracking without the interference of non-specific biological activation. Fluorescent stains including 4′,6-diamidino-2-phenylindole (DAPI), MitoTracker Deep Red FM, and LysoTracker Green DND-26 were acquired from Thermo Fisher Scientific (Waltham, MA, USA). For biochemical assessments, the Cell Counting Kit-8 (CCK-8) was obtained from Dojindo Molecular Technologies (Kumamoto, Japan), and the ATP Assay Kit, JC-1 Mitochondrial Membrane Potential Assay Kit, BCA Protein Assay Kit, and Lipid Peroxidation (MDA) Assay Kit were purchased from Beyotime Biotechnology (Shanghai, China). Commercial kits for the detection of Blood Urea Nitrogen (BUN) and Creatinine (Cr) were sourced from Nanjing Jiancheng Bioengineering Institute (Nanjing, China). Primary antibodies against CD63 (ab134045), CD81 (ab109201), TSG101 (ab125011), Calnexin (ab22595), Occludin (ab216327), and GAPDH (ab8245) were purchased from Abcam (Cambridge, UK), while HRP-conjugated and Alexa Fluor-conjugated secondary antibodies were obtained from Cell Signaling Technology (Danvers, MA, USA).

### 5.2 Cell culture

Human Renal Proximal Tubular Epithelial Cells (HK-2) were obtained from the American Type Culture Collection (ATCC; Manassas, VA, USA) and cultured in Dulbecco's Modified Eagle Medium/Nutrient Mixture F-12 (DMEM/F-12) supplemented with 10% fetal bovine serum (FBS, Gibco) and 1% penicillin/streptomycin in a humidified incubator at 37 °C with 5% CO_2_. For the isolation of mesenchymal stem cells (MSCs), bone marrow was flushed from the femurs and tibias of 4-week-old C57BL/6 mice with sterile PBS. The resulting cell suspension was cultured in α-MEM supplemented with 10% FBS, and non-adherent cells were removed after 24 h. MSCs were passaged upon reaching confluency, and cells from passages 3–5 were used for exosome isolation after phenotypic verification by western blot for positive markers (CD29, CD90, CD44).

### 5.3 Isolation and purification of MSC-derived exosomes

Exosomes were isolated using a standard differential ultracentrifugation protocol. Briefly, when MSCs reached approximately 80% confluency, the culture medium was replaced with exosome-depleted FBS medium (previously ultracentrifuged at 100,000×*g* for 18 h). After 48 h of incubation, the conditioned medium (CM) was collected and subjected to sequential centrifugation at 4°C to remove cellular debris: 300×*g* for 10 min, 2000×*g* for 20 min, and 10,000×*g* for 30 min. The supernatant was then filtered through a 0.22 μm PVDF filter to eliminate particles larger than 200 nm. Exosomes were pelleted by ultracentrifugation at 100,000×*g* for 70 min using a Beckman Coulter Optima XPN-100 Ultracentrifuge with a Type 45 Ti rotor. The resulting pellet was washed with sterile PBS and centrifuged again at 100,000×*g* for 70 min. The final purified exosome pellet was resuspended in PBS, quantified using a BCA Protein Assay Kit, and stored at −80 °C until further use.

### 5.4 Synthesis of Lipoic Acid-loaded ROS-responsive liposomes

Synthetic cubosome cores were prepared via a microfluidic assembly strategy. To form the organic phase, the cubic phase-forming lipid Phytantriol, the stabilizer DMG-PEG, the ROS-responsive probe (5 mol% relative to total lipids), and Lipoic Acid (drug-to-lipid weight ratio 1:5) were completely dissolved in absolute ethanol. The aqueous phase consisted of pure water. The organic and aqueous phases were continuously pumped into a microfluidic mixing chip using programmable syringe pumps to generate the Lipoic Acid-loaded cubosome cores. Specifically, the assembly was performed using a staggered herringbone micromixer (SHM) chip with main channel dimensions of approximately 200 μm (width) × 70 μm (depth). The flow rate ratio (FRR) of the aqueous phase to the organic phase was strictly maintained at 3:1, with an optimized total flow rate (TFR) of 2.0 mL/min. The entire primary synthesis process was conducted at a controlled temperature of 25 °C to ensure the uniform self-assembly of the cubic lipid phase. The resulting cubosome suspension was subsequently transferred to a dialysis bag (MWCO 3.5 kDa) and dialyzed against PBS for 24 h to completely remove the ethanol solvent and any unencapsulated Lipoic Acid.

### 5.5 Fabrication of membrane-fused EXLNPs via freeze-thaw-extrusion

The bio-synthetic hybrid EXLNPs were constructed using a non-destructive, sequential microfluidic fusion strategy. The freshly prepared synthetic LA-loaded cubosomes were co-injected with purified MSC-derived exosomes into a secondary microfluidic mixing stage. The exosomes and cubosomes were mixed at a pre-optimized protein-to-lipid weight ratio of 1:1. The total flow rate during this second mixing stage was strictly reduced to 200 μL/min (a 10-fold reduction compared to the initial cubosome formation step) to effectively minimize detrimental shear stress. The flow rate ratio between the exosome suspension and the cubosome suspension was set to 1:1. Furthermore, this controlled co-incubation and fusion process was carried out at a constant temperature of 37 °C to increase lipid membrane fluidity and preserve the native protein conformation of the exosomal surface membranes. Crucially, to preserve the biological integrity and native conformation of the exosomal surface membranes, the total flow rate during this second mixing stage was reduced by 10-fold compared to the initial cubosome formation step, effectively minimizing detrimental shear stress. During this controlled co-incubation, the high surface energy and intrinsic curvature of the synthetic cubosomes drove the spontaneous spreading and fusion of the exosomal membrane around the cubic core. The resulting membrane-fused EXLNPs were collected, stored at 4 °C, and utilized for subsequent in vitro and in vivo experiments within 3 days to maintain structural integrity.

### 5.6 Physicochemical characterization

The structural morphology of exosomes, liposomes, and EXLNPs was visualized using Transmission Electron Microscopy (TEM); samples were adsorbed onto glow-discharged carbon-coated copper grids, negatively stained with 2% phosphotungstic acid or uranyl acetate, and imaged on a JEOL JEM-1400 TEM at 80 kV. Hydrodynamic diameter (Z-average), Polydispersity Index (PDI), and Zeta potential were measured in triplicate using Dynamic Light Scattering (DLS) on a Zetasizer Nano ZS90 (Malvern Instruments) at 25 °C. Colloidal stability was assessed by monitoring particle size changes over 7 days in PBS and serum-containing medium. Protein marker retention was verified by Western Blot analysis, where lysed samples were separated by SDS-PAGE, transferred to PVDF membranes, and probed for exosomal markers (CD63, CD81, TSG101) and the ER marker Calnexin. The Encapsulation Efficiency (EE%) and Drug Loading (DL%) of Lipoic Acid were determined by High-Performance Liquid Chromatography (HPLC) after lysing the vesicles with methanol, using standard equations based on the weight of encapsulated drug versus total drug added or total nanoparticle weight, respectively.

### 5.7 In vitro ROS-responsive behavior and drug release

The fluorescence activation of EXLNPs was evaluated by incubating the nanoparticles (100 μg/ml) with varying concentrations of H_2_O_2_ (0–500 μM) in PBS, and fluorescence spectra were recorded using a fluorescence spectrophotometer (Hitachi F-7000). The in vitro release profile of Lipoic Acid was studied using the dialysis method, wherein EXLNPs in dialysis bags (MWCO 3.5 kDa) were immersed in release medium (PBS) containing varying concentrations of H_2_O_2_ (0 or 100 μM) and at different pH values (7.4 or 6.5) to mimic the AKI microenvironment. The setup was shaken at 37 °C, and at predetermined time points up to 48 h, aliquots were withdrawn for HPLC quantification to generate cumulative release curves.

### 5.8 Cellular uptake, lysosomal escape, and cytotoxicity

To investigate cellular interactions, HK-2 cells were treated with DiD-labeled EXLNPs (50 μg/ml) for 1, 2, and 4 h, fixed with 4% paraformaldehyde, stained with DAPI, and imaged using a Confocal Laser Scanning Microscope (CLSM, Leica TCS SP8). For intracellular trafficking analysis, cells treated with fluorescent EXLNPs were co-stained with LysoTracker Green DND-26 (50 nM) or MitoTracker Deep Red FM (100 nM); co-localization was quantified using ImageJ software to calculate the Pearson's Correlation Coefficient (PCC), assessing lysosomal escape and mitochondrial targeting. The biocompatibility of the nanocarriers was assessed using the CCK-8 assay, where HK-2 cells were incubated with increasing concentrations of EXLNPs (0–200 μg/ml) for 24 h, and cell viability was calculated relative to untreated controls.

### 5.9 Establishment of in vitro AKI model (hypoxia/reoxygenation)

To mimic ischemia-reperfusion injury in vitro, HK-2 cells were subjected to Hypoxia/Reoxygenation (H/R) conditions. Cells were cultured in glucose-free and serum-free medium within a hypoxia chamber maintained at 1% O_2_, 5% CO_2_, and 94% N_2_ for 12 h to induce hypoxia. Subsequently, the medium was replaced with full culture medium, and cells were returned to normoxic conditions (21% O_2_) for 6 h to simulate reoxygenation. Experimental groups included a normoxic Control, a PBS-treated H/R group, an Exosome-treated H/R group, and an EXLNP-treated H/R group, allowing for comparative assessment of therapeutic efficacy.

### 5.10 Assessment of mitochondrial bioenergetics (Seahorse analysis)

Mitochondrial respiration was monitored in real-time using the Seahorse XF Cell Mito Stress Test Kit on a Seahorse XFe24 Analyzer (Agilent Technologies). HK-2 cells were seeded in XFe24 microplates and subjected to H/R injury and respective treatments. Prior to the assay, the medium was replaced with Seahorse XF DMEM (pH 7.4) supplemented with 10 mM glucose, 1 mM pyruvate, and 2 mM glutamine, and cells were equilibrated in a CO_2_ incubator for 1 h. The Oxygen Consumption Rate (OCR) was measured at baseline and following sequential injections of Oligomycin (1.5 μM, ATP synthase inhibitor), FCCP (1.0 μM, uncoupler), and Rotenone/Antimycin A (0.5 μM, Complex I/III inhibitors). Key parameters including Basal Respiration, ATP Production, and Maximal Respiration were calculated by subtracting non-mitochondrial respiration rates from the corresponding measurement phases.

### 5.11 Measurement of mitochondrial membrane potential and ATP levels

Mitochondrial membrane potential (ΔΨՠ) was detected using the JC-1 probe; cells were stained with JC-1 working solution for 20 min, and the ratio of red fluorescence (aggregates in healthy mitochondria) to green fluorescence (monomers in damaged mitochondria) was analyzed by fluorescence microscopy to indicate ΔΨՠ levels. Intracellular ATP levels were quantified using a luciferase-based ATP Assay Kit, where cell lysates were mixed with the ATP detection working solution, and luminescence was measured using a microplate reader. All ATP concentrations were normalized to the total protein concentration of the respective samples to account for variations in cell number.

### 5.12 Evaluation of epithelial barrier integrity

To evaluate barrier function, HK-2 cells were seeded on Transwell inserts (0.4 μm pore size) to form a confluent monolayer, and Transepithelial Electrical Resistance (TEER) was measured using an epithelial volt-ohm meter (EVOM2) before injury and at 0, 6, 12, and 24 h post-treatment. Structural integrity was assessed by immunofluorescence staining; fixed and permeabilized cells were incubated with primary antibodies against Occludin overnight, followed by Alexa Fluor 488-conjugated secondary antibodies, and imaged via CLSM. Additionally, gene expression was quantified by Real-Time PCR (qPCR); total RNA was extracted using Trizol, reverse transcribed to cDNA, and amplified using SYBR Green Master Mix on an Applied Biosystems 7500 system. Relative expression of Occludin was calculated using the 2^−ΔΔCt^ method with GAPDH as the internal control.

### 5.13 RNA extraction and transcriptome sequencing (RNA-seq)

Total RNA was extracted from HK-2 cells in the Control, Model (H2O2), and Treatment (LNP) groups using Trizol reagent. RNA integrity and concentration were assessed using the Agilent 2100 Bioanalyzer. Library construction and eukaryotic mRNA sequencing were performed on the Illumina NovaSeq 6000 platform.

### 5.14 Bioinformatic analysis

Raw data were filtered to obtain clean reads, which were aligned to the reference genome using HISAT2. Gene expression levels were quantified as Fragments Per Kilobase of transcript per Million mapped reads (FPKM). Differential expression analysis was performed using the DESeq2 R package. Genes with an adjusted P-value (padj) < 0.05 and log_2> 1were identified as differentially expressed genes (DEGs). GO and KEGG pathway enrichment analyses were conducted using the clusterProfiler package. Pearson correlation analysis was performed to evaluate the relationship between metabolic markers and structural genes.

### 5.15 Animals and in vivo AKI model (ischemia-reperfusion)

All animal procedures were approved by the Institutional Animal Care and Use Committee (IACUC) and conducted in accordance with NIH guidelines. Male C57BL/6 mice (8–10 weeks old) were anesthetized, and a dorsal incision was made to expose the kidneys. The renal pedicles were dissected and clamped with non-traumatic microvascular clips to induce bilateral renal pedicle clamping for specific durations: 10, 20, or 40 min for the diagnostic correlation study, and 30 min for the therapeutic efficacy study. Body temperature was maintained at 37 °C during the procedure, and after clamp removal to allow reperfusion, the incision was sutured. Sham-operated mice underwent the same surgical procedure without pedicle clamping.

### 5.16 In vivo real-time theranostic imaging

To evaluate the diagnostic capability, AKI mice subjected to varying ischemia times and Sham mice were intravenously injected with EXLNPs (10 mg/kg lipid equivalent) via the tail vein. At predetermined time points (0, 30 min, 1 h, 4 h, 12 h, 24 h), mice were anesthetized and imaged using the IVIS Spectrum In Vivo Imaging System. The Total Radiant Efficiency in the kidney Region of Interest (ROI) was quantified to correlate fluorescence intensity with injury severity. Additionally, at 24 h post-injection, major organs (heart, liver, spleen, lung, kidney) were harvested for ex vivo fluorescence imaging to assess the homeand renal accumulation of the nanoparticles.

### 5.17 In vivo therapeutic regimen and assessment

Mice were randomly divided into four groups (n = 6): Sham, PBS (Vehicle), Exosome, and EXLNP. Treatments were administered intravenously immediately upon reperfusion. Renal function was monitored by measuring serum Blood Urea Nitrogen (BUN) and Creatinine (Cr) levels on Day 1 and Day 3 post-injury using commercial colorimetric kits. For histological analysis, kidneys harvested on Day 3 were fixed, embedded in paraffin, and sectioned for H&E staining, TUNEL assay, and CLDN 1 and 15 immunohistochemistry (IHC). Tubular injury was quantified using a Tubular Injury Score (0–4) based on the percentage of tubules showing necrosis, cast formation, or brush border loss, with at least 10 random fields evaluated per sample.

### 5.18 Statistical analysis

All quantitative data are presented as Mean ± Standard Deviation (SD). Statistical analysis was performed using GraphPad Prism 9.0 software. Differences between two groups were analyzed using the two-tailed Student's t-test, while comparisons among multiple groups were performed using One-way Analysis of Variance (ANOVA) followed by Tukey's post-hoc test. A p-value <0.05 was considered statistically significant (∗*p* < 0.05, ∗∗*p* < 0.01, ∗∗∗*p* < 0.001, ∗∗∗∗*p* < 0.0001).

## Funding

National Natural Science Foundation of China (82072174).

Grants from the National Key R&D Program of China (No. 2020YFA0908100).

CAS Key Laboratory of Interfacial Physics and Technology (CASKL-IPT1702).

## CRediT authorship contribution statement

**Fan Wu:** Formal analysis, Software, Validation. **Yi Shen:** Formal analysis, Project administration, Software. **Liang Dong:** Methodology, Resources. **Wei Nie:** Investigation, Supervision, Writing – original draft. **Wei Xue:** Funding acquisition, Resources, Supervision, Validation.

## Declaration of competing interest

We would like to submit the enclosed manuscript entitled “**A Membrane-Fused Theranostic Nanoplatform for Real-Time ROS Imaging and Mitochondrial Resuscitation-Driven Barrier Repair in Acute Kidney Injury**”, which we wish to be considered for possible publication as an **article** in **Materials Today Bio**.

The co-authors listed have seen and agree with the content of the manuscript and there is no financial or proprietary interest in any material or method mentioned. We certify that the submission is original work that has not been published previously, and not under consideration for publication elsewhere, in whole or in part. We wish this paper could be evaluated as soon as your convenience. Thank you very much!

## Data Availability

Data will be made available on request.
