# Supplementary material for: A membrane-fused theranostic nanoplatform for real-time ROS imaging and mitochondrial resuscitation-driven barrier repair in acute kidney injury
Source: Mater Today Bio. 2026 May 13;38:103217. doi: 10.1016/j.mtbio.2026.103217 (PMC13240824; doi:10.1016/j.mtbio.2026.103217)
Supplement: Multimedia component 1 [file mmc1.docx]

Supplementary Materials for

**A Membrane-Fused Theranostic Nanoplatform for Real-Time ROS Imaging and Mitochondrial Resuscitation-Driven Barrier Repair in Acute Kidney Injury**

Fan Wu ^a^, Yi Shen ^b^, Liang Dong ^a^, Wei Nie ^c, d^*, Wei Xue ^a^*

a. Department of Urology, Ren Ji Hospital, Shanghai Jiao Tong University School of Medicine, Shanghai, 200120, China.

b. Shanghai Center for Systems Biomedicine, Key Laboratory of Systems Biomedicine (Ministry of Education), Shanghai Jiao Tong University, Shanghai 200240, China

c. Affiliated Hospital 6 of Nantong University, Yancheng 224001, China.

d. Wake Forest Institute for Regenerative Medicine, Wake Forest School of Medicine, Winston Salem 27103, NC, USA

*Corresponding author

Prof. Wei Nie, Affiliated Hospital 6 of Nantong University, Yancheng 224001, China; Wake Forest Institute for Regenerative Medicine, Wake Forest School of Medicine, Winston-Salem, NC 27103, USA. Email: webernelson@163.com

Professor Wei Xue, Department of Urology, Ren Ji Hospital, Shanghai Jiao Tong University School of Medicine, Shanghai, 200120, China. [xuewei@renji.com](mailto:xuewei@renji.com).

**This file includes:**

Figs. S1 to S4


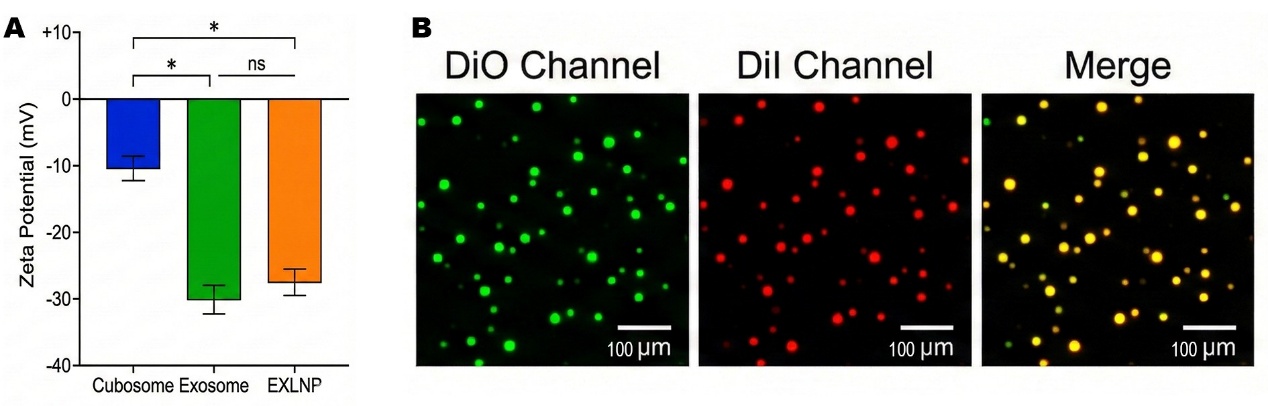


**Fig. S1.** (A) Zeta potential measurements of synthetic Cubosomes, purified MSC-derived Exosomes, and hybrid EXLNPs. The EXLNP group exhibits a negative surface charge (approximately -24.5 mV) that closely mirrors that of native Exosomes (approximately -22.1 mV), distinctly different from the synthetic Cubosome cores (approximately -8.4 mV). This charge similarity confirms that the outer surface of the hybrid nanovesicles is dominated by the exosomal membrane, validating the successful membrane fusion and coating process.


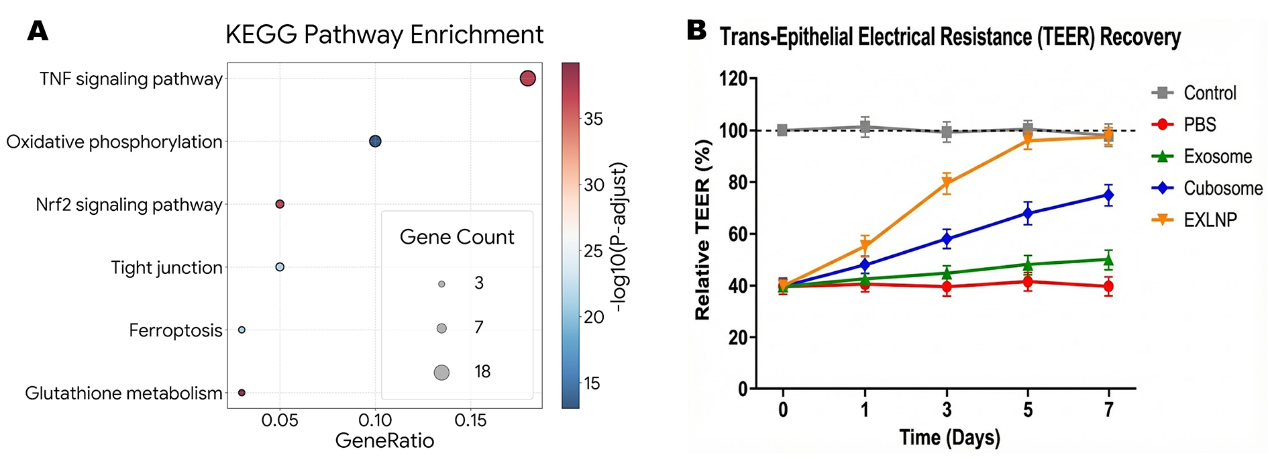


**Fig. S2.** (A) Kyoto Encyclopedia of Genes and Genomes (KEGG) pathway enrichment analysis was performed on the differentially expressed genes to identify key biological processes. Significant enrichment in "Tight junction," "Oxidative phosphorylation," and "Metabolic pathways" was observed, confirming that the restoration of barrier structure is genetically coupled to mitochondrial bioenergetics. (B) The functional integrity of the epithelial barrier was assessed by monitoring the Transepithelial Electrical Resistance (TEER) of HK-2 monolayers over time. A rapid and sustained recovery of electrical resistance was recorded in the EXLNP group, indicating that the paracellular seal was effectively re-established, whereas resistance remained suppressed in the PBS-treated group.


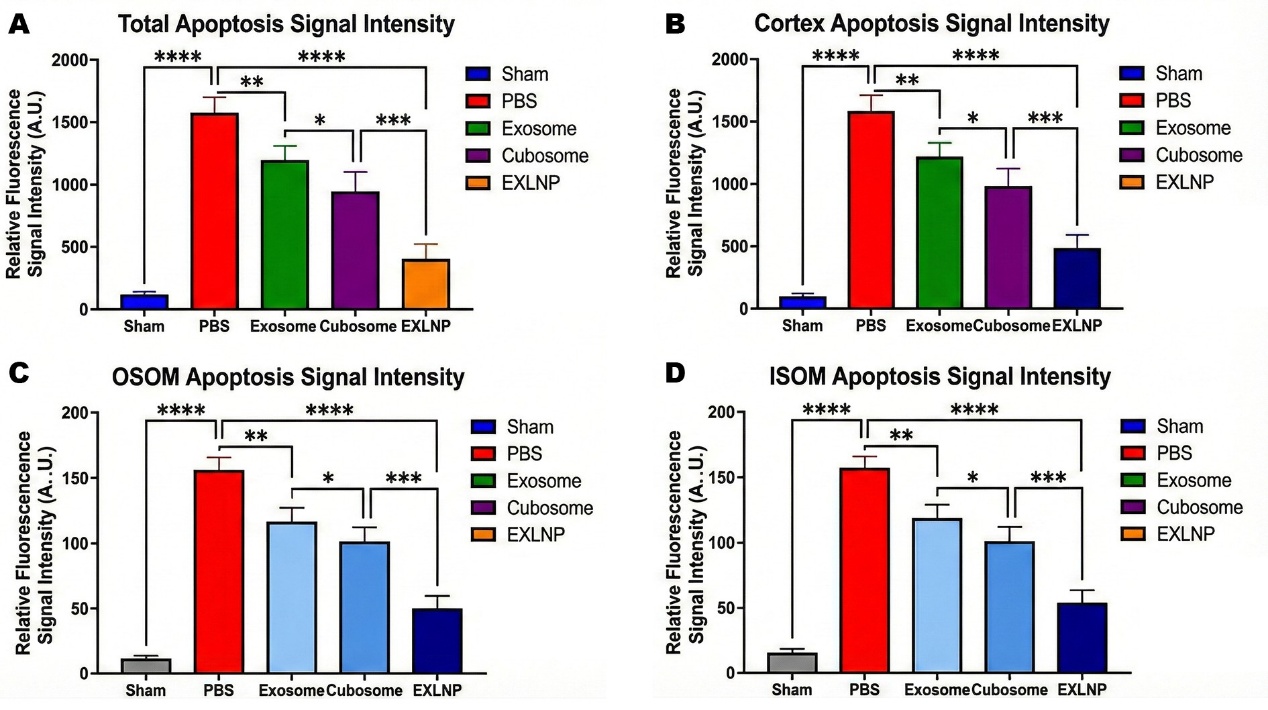


**Fig. S3.** (A) Quantification of the total fluorescence signal intensity derived from TUNEL staining across all kidney regions. The EXLNP group exhibits a robust reduction in overall apoptosis compared to the PBS and single-component control groups. (B–D) Region-specific quantitative analysis of apoptotic signals in the (B) Cortex, (C) Outer Stripe of Outer Medulla (OSOM), and (D) Inner Stripe of Outer Medulla (ISOM). In all analyzed anatomical regions, the highest apoptosis intensity was observed in the PBS group. Conversely, EXLNP treatment consistently suppressed apoptotic signaling to levels significantly lower than those of the Exosome and Cubosome groups, particularly in the highly vulnerable OSOM and ISOM regions.


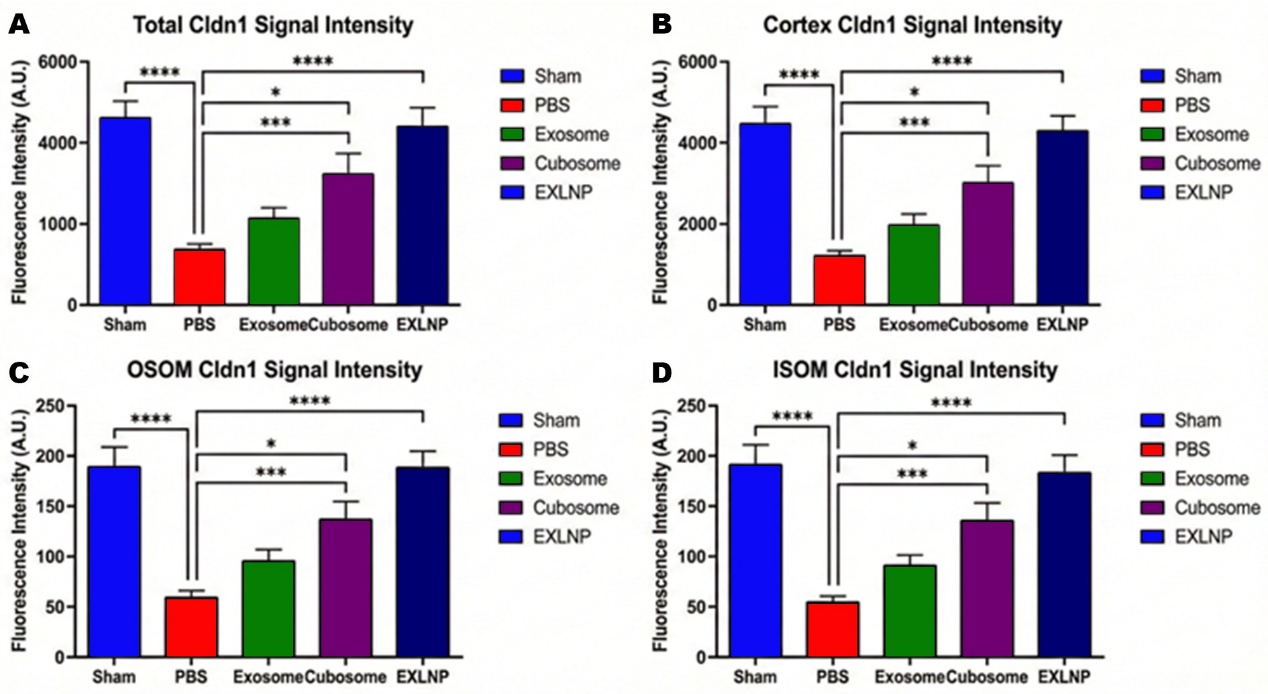


**Fig. S4.** (A) Quantification of the total immunofluorescence signal intensity of CLDN1 across the whole kidney section. The PBS-treated group exhibits a severe loss of CLDN1 protein expression, whereas EXLNP treatment restores expression levels to a magnitude comparable to the healthy Sham group.(B–D) Region-specific quantitative analysis of CLDN1 intensity in the (B) Cortex, (C) Outer Stripe of Outer Medulla (OSOM), and (D) Inner Stripe of Outer Medulla (ISOM). In all anatomical regions, a distinct hierarchy of recovery was observed: while native Exosomes provided modest improvement, the Cubosome group exhibited significantly higher CLDN1 expression than the Exosome group (*p* < 0.05 or *p* < 0.001), suggesting that the delivery of antioxidant Lipoic Acid facilitates tight junction re-assembly. However, the EXLNP group consistently achieved the highest expression levels, confirming the synergistic efficacy of the hybrid nanovesicles in restoring the epithelial barrier.
